# A Role of DLPFC in the Learning Process of Human Mate Copying

**DOI:** 10.3389/fpsyg.2016.00546

**Published:** 2016-04-19

**Authors:** Jin-Ying Zhuang, Jiajia Xie, Die Hu, Mingxia Fan, Li Zheng

**Affiliations:** ^1^Shanghai Key Laboratory of Brain Functional Genomics, School of Psychology and Cognitive Science, East China Normal UniversityShanghai, China; ^2^Shanghai Key Laboratory of Magnetic Resonance, Department of Physics, East China Normal UniversityShanghai, China; ^3^Key Laboratory of Brain Functional Genomics, Ministry of Education, Shanghai Key Laboratory of Brain Functional Genomics, School of Psychology and Cognitive Science, East China Normal UniversityShanghai, China

**Keywords:** mate copying, social learning, cognitive execution, fMRI, DLPFC

## Abstract

In the current study, we conducted a behavioral experiment to test the mate coping effect and a functional magnetic resonance imaging (fMRI) experiment to test the neural basis involved in the social learning process of mate copying. In the behavioral experiment, participants were asked to rate the attractiveness of isolated opposite-sex (potential mates) facial photographs, then shown the targets associating with a neutral-faced model with textual cues indicating the models’ attitude (interested vs. not-interested) toward the potential mates, and then asked to re-evaluate the potential mates’ attractiveness. Using a similar procedure as the behavioral experiment, participants were scanned while observing the compound images in the fMRI experiment. The mate copying effect was confirmed in the behavioral experiment –greater increase in attractiveness ratings was observed for opposite-sex photographs in the interested than in the not-interested condition. The fMRI results showed that the dorsolateral prefrontal gyrus (DLPFC) was significantly active in the comparison of interested > not-interested condition, suggesting that a cognitive integration and selection function may be involved when participants process information from conditions related to mate copying.

## Introduction

When it comes to assessing the attractiveness of men, women can be influenced by other women relatively easily. For example, in a study by Eva and Wood (2006), in which 38 women rated physical attractiveness of men in photographs, men who were identified as married were generally rated as more physically attractive than men who were identified as single. Women rated men as being more desirable if the men were pictured with other women rather than pictured alone or with other men (Hill and Buss, 2008). This social transmission of mate preferences has been broadly referred to as mate copying or mate-choice copying and has been described in humans in recent years (Eva and Wood, 2006; Jones et al., 2007; Waynforth, 2007; Little et al., 2008; Place et al., 2010; Yorzinski and Platt, 2010; Bowers et al., 2011).

The attitude of the same-sex partner (the model) toward a potential mate (the target) has been shown to influence observers’ assessment intensively (Jones et al., 2007; Place et al., 2010; Yorzinski and Platt, 2010). For example, Jones et al. (2007) performed a study in which the participants viewed the same males associating with either a female with smiling face (showing interest) or a female with a neutral expression (not showing interest). They found that observing a paired female showing interest in the male enhanced observers’ preference for that male to a greater extent than did observing a paired female with an uninterested expression. Although it has been observed more in women, mate copying effect has also been systematically observed in men (Place et al., 2010; Yorzinski and Platt, 2010). For example, both men and women were observed to express more interest in engaging in a relationship with a potential mate if that mate was paired with a partner with smiling face (Yorzinski and Platt, 2010).

Mate copying has been hypothesized to save time and cognitive effort through social learning otherwise needed to evaluate the quality of potential mates (Dugatkin, 1992; Pruett-Jones, 1992; Westneat et al., 2000). However, individual-based copying also carries a possibly costly consequence: It places mate seekers especially in the thick of competition (Brennan et al., 2008), specifically when the mates have already been chosen by others. In many species, competition often leads to intra-sexual conflict which can induce serious survival costs (Bowers et al., 2011). Therefore, some researchers have suggested that mate copying is a strategic use of public information via social learning (Richerson and Boyd, 2005; Little et al., 2008, 2010). That is, mate copying may be not just a blind copying, but a process involving copiers’ recon on trade-offs between personal and public information use (Laland, 2004; Kendal et al., 2005), and how and when the public information will be most useful (Little et al., 2010). Indeed, once social learning evolved, it would pay to be selective about whom to learn from and what cues to learn because some models will be more successful than others (Henrich and Gil-White, 2001). Previous studies have shown this ‘selection bias’ in mate copying. For example, men and women were influenced in their judgment of attractiveness of potential mates by the apparent choice of attractive members of the same sex (Sigall and Landy, 1973; Yorzinski and Platt, 2010; Little et al., 2011). Another study using images that were presented with a fictitious partner has shown that both men and women find a face paired with an attractive partner to be more attractive than one paired with an unattractive partner for a long-term but not a short-term relationship (Little et al., 2008). In non-human species, bias has been observed in fish that while younger female guppies copy the choice of older females, the latter do not copy the choice of the former (Dugatkin and Godin, 1993).

In addition, copying effects can be trait-based and more general, where individuals learn about the traits of those chosen and find those traits more attractive in other individuals (White and Galef, 2000; Godin et al., 2005; Swaddle et al., 2005). This generalization has also been shown quite complicated. For example, while the generalized change of attractiveness ratings has been found with manipulations of eye-spacing (Little et al., 2010), the other studies indicate that both males and females fail to exhibit trait-based mate copying for facial traits, yet exhibit it for hair and clothing traits (Bowers et al., 2011) and shirt color trait (Place et al., 2010).

In a summary, in the learning process of mate copying, copiers may need to integrate and select information from the observed partners and then make an appropriate response accordingly. This cognitive execution function has been found especially related to the dorsolateral prefrontal cortex (DLPFC) in recent study (Buckholtz and Marois, 2012). For example, the ‘integration-and-selection’ hypothesis suggests that the role of DLPFC is to guide the selection of a specific response from among possible response options by integrating relevant information with context-specific rules about how to apply this information (Buckholtz and Marois, 2012). Thus, we hypothesized that compared with the condition of models with uninterested attitude toward the target, the DLPFC should be more active in the condition where models are interested in the potential mates because copiers have to integrate information to recon on trade-offs between personal and public information use (Laland, 2004; Kendal et al., 2005) and which information will be most useful to make an adaptive decision.

Accordingly, we conducted a behavioral and a fMRI experiment to test our hypothesis. The behavioral experiment was conducted to test the effect of mate copying by using a similar paradigm of previous research (Little et al., 2008; Place et al., 2010; Yorzinski and Platt, 2010; Bowers et al., 2011). Specifically, participants were first asked to rate the attractiveness of isolated opposite-sex (potential mates) faces, then shown the targets associating with a neutral-faced model (compound images) with textual cues indicating the models’ attitudes (interested vs. not-interested) toward the potential mates, and then asked to re-evaluate the potential mates’ attractiveness. The effect of mate copying was assessed by calculating the attractiveness ratings assigned to the individual opposite-sex facial photographs before vs. after viewing the compound images in the interested and not-interested conditions. The fMRI experiment was conducted using the similar rating-observation-re-rating procedure. The scanning process was performed while participants viewing the compound images, which has been the key social learning process in mate copying (Richerson and Boyd, 2005; Jones et al., 2007; Little et al., 2008, 2010, 2011; Yorzinski and Platt, 2010; Bowers et al., 2011). The activated brain regions involved in the learning process related to mate copying can be extracted by comparing trials in the interested condition with trials in the not-interested condition. The difference in DLPFC activation was predicted to be captured by the above-mentioned statistical analyses.

## Materials and Methods

### Participants

There were 92 participants (46 of each sex; age range: 18–24 years; mean age ± standard deviation: 21.56 ± 2.12 years) recruited from a university community with flyers and by word of mouth. All were healthy, self-reported heterosexuals with normal or corrected-to-normal vision, and all provided written informed consent. The study protocols were approved by the ethics committee of the university. Participants were randomly assigned to separate behavioral (*N* = 60, 30 of each sex) or scanning (*N* = 32; 16 of each sex) pools at signup. Scanning participants were screened for current psychiatric diagnoses, and right-handedness. Participants were paid and debriefed after they finished the experiment.

### Stimuli

#### Individual Photographs

Stimuli were color photographs of 64 men and 64 women from the local university student population. All photographs captured a neutral expression and showed a frontal view without makeup, accessories, or glasses. They were cropped at the neck and adjusted to 300 × 300 pixels against a white background in Adobe Photoshop. The lighting conditions were adjusted to a consistent standard. The photographs were rated for attractiveness by other participants from the university using a 1–7 Likert scale (1 = very unattractive, 7 = very attractive). The rater group included 30 males and 35 females (age range, 18–27 years). Mean attractiveness scores for male and female faces were 3.06 ± 0.80 and 3.32 ± 0.78, respectively. We included photographs with mid-range attractiveness scores (*M* ± 1 *SD*) to exclude extremely attractive or unattractive individuals. Neither the participants who rated the photographs nor the individuals in the photographs participated in the behavioral/fMRI experiments.

#### Compound Images

**FIGURE 1:**
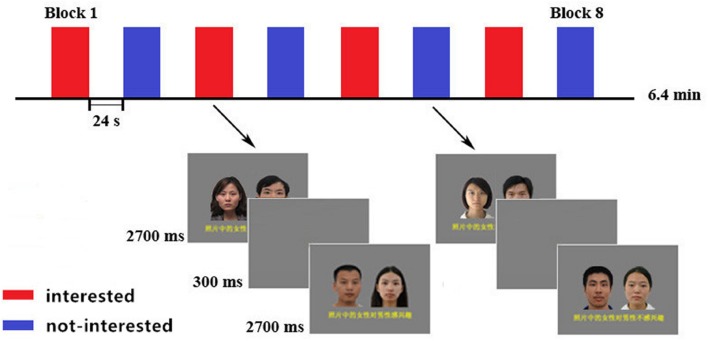
**Compound images and Procedure of the fMRI experiment.** Block design with four blocks for each condition. Each block contained eight compound images of the same type. Two kinds of blocks were alternated between each other, with the order of the blocks counterbalanced across participants. A black fixation cross appeared in the center of the screen during the 24-s pre-block rest periods.

Photographs were edited in Adobe Photoshop to create dual-image compounds (300 × 400 pixels). Each compound image included one male and one female photograph, which were chosen randomly and arranged side by side against a gray background (**Figure 1**). The side on which the female face was displayed was counterbalanced across the compounds. In total, 64 compound images were used in the experiment.

#### Textual Cues

All word cues were in Simplified Chinese and placed at the bottom of the compound images. An affirmative sentence “
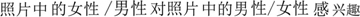
” (The woman/man in the picture is interested in the man/woman) was assigned to half of the compounds, and the annulling sentence “
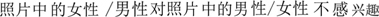
” (The woman/man in the picture is not interested in the man/woman) was assigned to the other half of the compounds. For each sex of the participants, they would only be presented with words indicating the same-sex models’ attitude toward the opposite-sex potential mates.

### Procedure

A rating-observation-re-rating procedure was performed. The participants performed the whole process at a computer and finished the re-rating process immediately after each compound image in the behavioral experiment, whereas participants performed the observation process in the scanner and completed the rating and re-rating processes immediately before and after scanning, respectively, outside of the scanner in the fMRI experiment. The task was otherwise identical.

Specifically, in the first rating process, each participant was shown the 64 individual opposite-sex photographs one by one, in a random order on a computer monitor and was asked to rate each face for attractiveness using the 1–7 Likert scale (where 1 = very unattractive, 7 = very attractive) on a self-determined pace.

For the behavioral participants, after the initial rating process, each of the 64 compound images was randomly presented for 2700 ms with an inter-stimulus interval (ISI) of 300 ms and the participants were asked to observe them attentively. As soon as the compound image disappeared, the target (the opposite-sex photograph) in the compound was presented and participants were asked to rate the attractiveness using the 1–7 Likert scale in a self-paced speed.

In the fMRI experiment, we scanned the observation process after the initial rating process out of the scanner. After a structural scan, each participant’s assigned series of image compounds was presented and they were asked to observe them attentively. The stimulus presentation, timing, and counterbalancing of the compound images are outlined in **Figure 1**. There were eight blocks. Each block contained eight instances of the same compound type. Each compound image was presented for 2700 ms with an inter-stimulus interval of 300 ms. Images of each type block (interested vs. not interested) were presented in an alternating fashion, with the presentation order of the blocks being counterbalanced across participants. Each block lasted for 24 s with a 24-s rest between blocks, during which a black fixation cross was presented against the gray background. The re-rating process was a repetition of the first one, which was performed immediately after the scanning outside of the scanner.

After finishing the experiment, all participants reported that they were not familiar with any of the individuals pictured.

### Data Acquisition

Imaging was performed with a 3.0-T Siemens Trio Tim Scanner (Erlangen, Germany) with a 32-channel head coil. T1-weighted sagittal structural images were acquired first with the following parameters: TR/TE = 2530 ms/2.34 ms, field of view (FOV) = 256 mm × 256 mm, flip angle = 7°, and voxel size = 1 mm × 1 mm × 1 mm, 192 slices. The stimuli were presented via the *in vivo* ESys fMRI system (Gainesville, FL, USA). Functional images were obtained with a gradient echo-planar imaging sequence (TR/TE = 2000 ms/30 ms, FOV = 192 mm × 192 mm, flip angle = 90°, voxel size: 3.13 mm × 3.13 mm × 3.5 mm, 32 slices).

### Data Analysis

#### Behavior

The dependent behavioral variable in the two experiments was the attractiveness rating that the participants assigned to the individual opposite-sex facial photographs. A 2 (pre- *vs*. post-observation) × 2 (interested *vs*. not-interested) repeated measures of ANOVA (rmANOVA) on the attractiveness rating of opposite-sex faces was conducted to obtain the effects of experimental manipulations on mate copying.

#### Functional Magnetic Resonance Imaging

Imaging data preprocessing was performed using Statistical Parametric Mapping software, version 8 (SPM8; The Wellcome Department of Imaging Neuroscience, London, UK). The first four volumes were discarded to exclude calibration effects. The functional images were realigned to the first image to correct for interscan head movements. Six participants (four male, two female) who had excessive head movement (translation ≥ 2 mm, rotation ≥ 2°) were excluded from further analysis. The individual T1-weighted, 3D structural image was co-registered to the mean EPI image generated after realignment. The co-registered structural image was then segmented into gray matter (GM), white matter (WM) and cerebrospinal fluid (CSF) using a unified segmentation algorithm (Ashburner and Friston, 2005). The functional images after the realignment procedure were spatially normalized to the Montreal Neurological Institute (MNI) space (resampled to 2 mm ^∗^2 mm ^∗^2 mm) using the normalization parameters estimated during unified segmentation and then spatially smoothed with a Gaussian kernel of 8 mm full-width half-maximum (FWHM).

Statistical analyses were performed using a general linear model. The two stimulus types (interested, not-interested) were modeled as a boxcar function convolved with the canonical hemodynamic response. The models additionally included six movement parameters derived from realignment as covariates of no interest. We applied a high-pass filter with a cut-off of 128 s to remove low-frequency signal components. For each subject at the first-level analysis, simple main effects for the interested and not-interested conditions were calculated by applying the ‘1 0’ contrasts. The two first-level individual contrast images were then analyzed at the second group level by employing the random-effects model.

The main effect of models’ attitude was calculated by contrasting trials in the interested condition with trials in the not-interested condition (interested > not-interested) and the reverse contrast (not-interested > interested) to identify brain regions involved in the learning process of mate copying. Activations were reported significant with a voxel-level threshold of *p* < 0.001 (uncorrected), and a cluster size of *k* > 50.

## Results

### Behavioral Results

**FIGURE 2:**
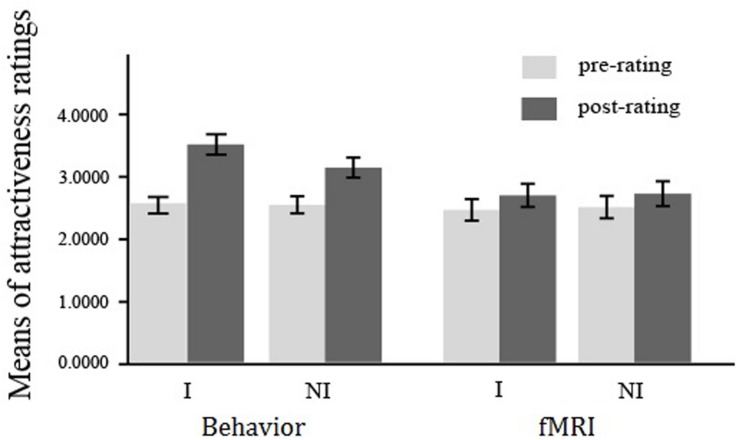
**Mean of attractiveness ratings in the behavioral and fMRI experiments.** Mean of attractiveness ratings shown for each stimulus type with error bars indicating the standard error of the mean (SEM). I, interested condition; NI, not-interested condition.

For the behavioral experiment, a 2 (pre- *vs*. post-observation) × 2 (interested *vs*. not-interested) repeated measures of ANOVA (rmANOVA) on the attractiveness rating of opposite-sex faces revealed significant main effects of pre-to-post ratings (*F*_1,59_ = 25.88, *p* < 0.001, $\eta_{p}^{2}$ = 0.31) and attitude (*F*_1,59_ = 53.28, *p* < 0.001, $\eta_{p}^{2}$ = 0.48). The effect of interaction was also significant, *F*_1,59_ = 25.32, *p* < 0.001, $\eta_{p}^{2}$ = 0.30. Simple effects analysis showed that though the post-attractiveness ratings were significantly higher than the pre-ratings for both conditions (interested: *M*_post_ = 3.49, *SD*_post_ = 1.26; *M*_pre_ = 2.54, *SD*_pre_ = 1.02; *F*_1,59_ = 66.10, *p* < 0.001, $\eta_{p}^{2}$ = 0.53; not-interested: *M*_post_ = 3.12, *SD*_post_ = 1.24; *M*_pre_ = 2.52, *SD*_pre_ = 1.06; *F*_1,59_ = 31.92, *p* < 0.001, $\eta_{p}^{2}$ = 0.35), the post attractiveness rating in the interested condition was significantly higher than that in the not-interested condition, *F*_1,59_ = 34.56, *p* < .001, $\eta_{p}^{2}$ = 0.37 (see **Figure 2**). These combining results confirmed a mate copying effect presented in the interested condition.

For the fMRI experiment, a 2 (pre- *vs*. post-observation) × 2 (interested *vs*. not-interested) repeated measures of ANOVA (rmANOVA) on the attractiveness rating of opposite-sex faces revealed significant main effect of pre-to-post ratings (*F*_1,25_ = 4.36, *p* < 0.048, $\eta_{p}^{2}$ = 0.15), but not of the attitude (*F*_1,25_ = 1.06, *p* = 0.31, $\eta_{p}^{2}$ = 0.04). The effect of interaction was also not reaching the significant level, *F*_1,25_ = 0.085, *p* = 0.77, $\eta_{p}^{2}$ = 0.004 (see **Figure 2**).

### fMRI Results

**FIGURE 3:**
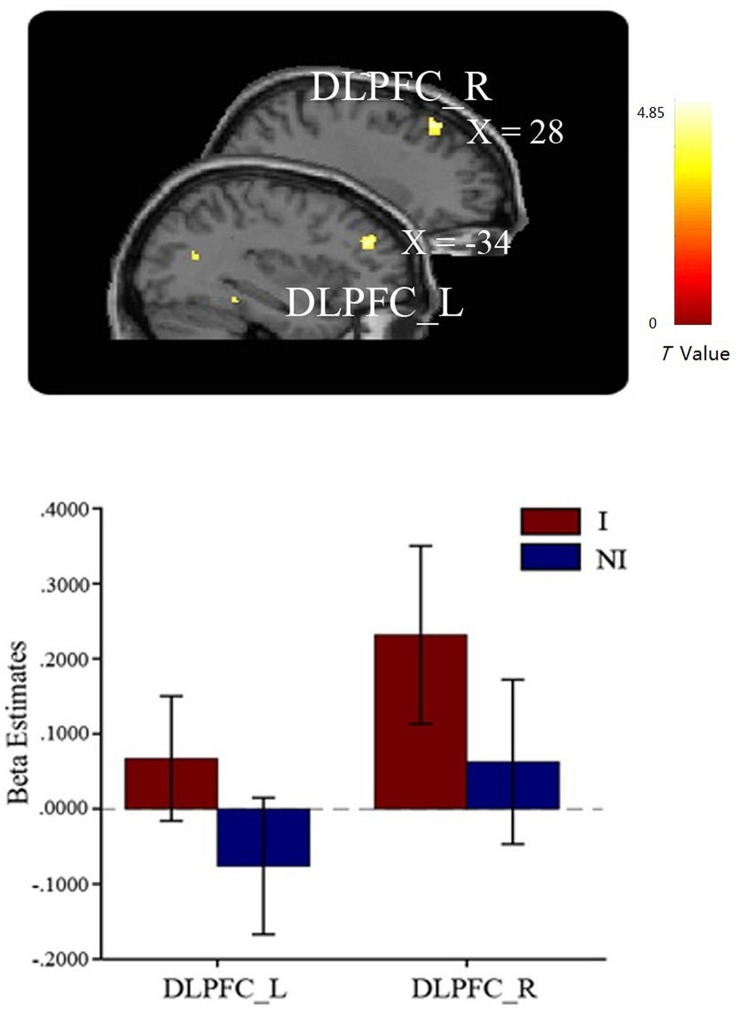
**Greater activations in bilateral DLPFC were found in the interested condition compared to the not-interested condition when participants viewing the compound images.** Error bars indicate one SEM. I, interested condition; NI, not-interested condition.

In the comparison of interested > not-interested condition, the bilateral DLPFCs were observed significantly activated (**Figure 3**), but not in the reverse comparison (not-interested > interested) (see **Table 1**). These results confirmed our hypothesis that the DLPFC should be involved in the learning process of mate copying.

**Table 1:** Regions associated with the effect of mate copying.

Regions of activation	Side	MNI coordinates			*T*-score	Voxels
				
		*X*	*Y*	*Z*		
**Interested > not-interested**						
Vermis	R	2	42	-32	5.05	128
Middle occipital gyrus	L	-42	-72	20	4.69	123
DLPFC	L	-34	40	26	4.85	75
	R	28	32	50	4.65	61
Middle temporal gyrus	L	-64	-54	10	4.42	68
Fusiform	L	-32	-40	-12	4.46	57
**Not-interested > interested**						
Inferior occipital gyrus	L	-28	-94	-6	4.71	304
Fusiform	R	26	-80	-2	4.09	248
Lingual	R	22	-82	-4	4.05	
Middle temporal gyrus	R	52	-74	8	4.49	168
Inferior occipital gyrus	R	48	-78	-6	4.23	
Inferior temporal gyrus	R	42	-66	-8	4.10	


*Coordinates (mm) are in MNI space. L, left, R, right. *p* < 0.001, uncorrected, *k* > 50. Sample size = 26.*

## Discussion

The present study examined the effect of mate copying while manipulating models’ attitude. The mate copying effect was confirmed in our behavioral experiment – greater increased attractiveness ratings for opposite-sex photographs were observed when they were paired with models with interested attitude than with models with not-interested attitude. However, the behavioral results of the fMRI experiment revealed no significant mate copying effect. The only difference between the two experiments was that the re-rating process was performed immediately after the observation of each compound image in the behavioral experiment, while during the fMRI study, participants observed all compound images in the scanner and then completed re-rating processes outside of the scanner. It may be indicated that mate copying did exist when observing models’ attitude to potential mates. When asked to re-rate outside of the scanner in the fMRI study, mate copying effect may be weakened due to the time delay and/or the environmental change.

The fMRI data confirmed our hypothesis that the DLPFC is involved in the social learning process in human mate copying. In detail, the bilateral DLPFC were more active when participants observed models with an interested attitude than with a not-interested attitude. The DLPFC has been shown involved in cognitive execution within working memory (Owen, 2000; du Boisgueheneuc et al., 2006) and attention (Fox et al., 2006). It has been found to be particularly involved in integrating information from social context to enable an appropriate social decision. For example, Spitzer et al. (2007) scanned participants (Player A) while they made decisions about how much of a monetary endowment to split with another, anonymous participant (Player B) and found that the DLPFC is essential for integrating information about sanction threats into decision making to incentivize norm-compliant behavior. The activation of DLPFC in the current study may reflect an involvement of cognitive execution function in the social learning process of mate copying in two aspects. First, it integrates and selects information from the observation, such as the social popularity and prestige of models, the mate values of the model and the target, or even how the model’s and target’s mate values compared to that of the observer (Vukomanovic and Rodd, 2007). Secondly, it guides to make an appropriate response in mate copying with the ability of DLPFC to maintain stable goal representations over time (Miller and Cohen, 2001).

## Conclusion

The present study provided a novel experimental investigation of neural basis involved in the social learning process of human mate copying. The DLPFC was significantly activated in the comparison of interested > non-interested condition, suggesting that a cognitive integration and selection function may be involved when participants process information from conditions related to mate copying.

## Author Contributions

J-YZ developed the study concept and design. Testing and data collection were performed by JX, DH, and MF. JX and DH performed the data analysis and interpretation under the supervision of LZ and J-YZ. J-YZ drafted the manuscript, and LZ provided critical suggestions. All authors approved the final version of the manuscript for submission.

## Conflict of Interest Statement

The authors declare that the research was conducted in the absence of any commercial or financial relationships that could be construed as a potential conflict of interest.
